# Porcine versus bovine surfactant therapy for RDS in preterm neonates: pragmatic meta-analysis and review of physiopathological plausibility of the effects on extra-pulmonary outcomes

**DOI:** 10.1186/s12931-019-1267-8

**Published:** 2020-01-07

**Authors:** Silvia Foligno, Daniele De Luca

**Affiliations:** 1 Division of Pediatrics and Neonatal Critical Care, Medical Center “A. Béclère”, Paris Saclay University Hospitals, Assistance Publique–Hôpitaux de Paris (APHP), Paris, France; 2 Division of Pediatrics and Neonatal Critical Care, Medical Center “A. Béclère”, Paris Saclay University Hospitals, Assistance Publique–Hôpitaux de Paris (APHP) and Paris-Saclay University, Paris, France

**Keywords:** Surfactant, Non-respiratory outcome, Plausibility, Therapy

## Abstract

**Background:**

While porcine seems to be superior to bovine surfactants in terms of respiratory outcomes, it is unclear if a surfactant can improve extra-pulmonary outcomes in preterm neonates with respiratory distress syndrome and if there is any physiopathological/biological mechanism linking surfactant therapy to these outcomes. We aim to fill these knowledge gaps.

**Methods:**

Systematic and pragmatic review coupled with meta-analysis of randomized controlled trials of bovine or porcine surfactants administered to treat RDS in preterm neonates; common extra-pulmonary neonatal intensive care outcomes were considered. As additional analysis, animal or human translational studies about mechanisms linking surfactant replacement to extra-pulmonary neonatal outcomes were also systematically reviewed.

**Results:**

Porcine surfactant is associated with lower incidence of patent *ductus arteriosus* (OR:0.655; 95%CI:0.460–0.931); *p* = 0.018; 12 trials; 1472 patients); prenatal steroids (coeff.:-0.009, 95%CI:-0.03–0.009, *p* = 0.323) and gestational age (coeff.:0.079, 95%CI:-0.18–0.34, *p* = 0.554) did not influence this effect size. No significant differences were found between porcine and bovine surfactants on neonatal intensive care unit length of stay (mean difference (days):-2.977; 95%CI:-6.659–0.705; *p* = 0.113; 8 trials; 855 patients), intra-ventricular hemorrhage of any grade (OR:0.860; 95%CI:0.648–1.139); *p* = 0.293; 15 trials; 1703 patients), severe intra-ventricular hemorrhage (OR:0.852; 95%CI:0.624–1.163); *p* = 0.313; 15 trials; 1672 patients), necrotizing entero-colitis (OR:1.190; 95%CI:0.785–1.803); *p* = 0.412; 9 trials; 1097 patients) and retinopathy of prematurity (OR:0.801; 95%CI:0.480–1.337); *p* = 0.396; 10 trials; 962 patients).

**Conclusions:**

Physiopathological mechanisms explaining the effect of surfactant have been found for patent *ductus arteriosus* only, while they are lacking for all other endpoints. Porcine surfactant is associated with lower incidence of PDA than bovine surfactants. As there are no differences in terms of other extra-pulmonary outcomes and no physiopathological plausibility, these endpoints should not be used in future trials.

**Registration:**

PROSPERO n.CRD42018100906.

## Introduction

Respiratory distress syndrome (RDS) represents the main cause of respiratory failure in preterm neonates and is associated with an increasing burden of care [1]. Since RDS is caused by primary surfactant deficiency, the availability of exogenous surfactants allows an effective replacement therapy, which is recommended by current international guidelines, in neonates failing continuous positive airway pressure (CPAP) [2, 3]. The combined use of CPAP early from birth and surfactant replacement has provided significant benefits in terms of mortality and broncho-pulmonary dysplasia (BPD) [4]. Meta-analyses have demonstrated that surfactant replacement is more effective if performed: 1) within the first 2-3 h of life [5], and 2) with current animal-derived surfactants preparations, rather than with older synthetic, protein-free surfactants [6]. Moreover, our recent meta-analysis demonstrated the superiority of high dose poractant-α over bovine surfactants *at their licensed dose* in terms of respiratory outcomes, using a pragmatic design [7]. This has been possible because earlier meta-analysis showed clinical equivalence between bovine surfactants [8] and this has a strong biological plausibility given their similar biochemical composition and pharmacological features [7, 9, 10]. The biochemical and pharmacological characteristics of different surfactant preparations are detailed in our previous work [7]. The beneficial effect of surfactant replacement on BPD and other respiratory outcomes is physiopathologically sound, as surfactant increases compliance and alveolar recruitment reducing the need for distending pressure and invasive mechanical ventilation, which is a main pro-inflammatory trigger involved in BPD development [11]. Surfactant replacement also stimulates the endogenous surfactant production [12] and reduces the incidence of air leaks [13], since the improved compliance allows an efficacious ventilation with a lower transpulmonary pressure.

However, it is not clear if surfactant can actually have any effect on extra-pulmonary outcomes [14, 15]. Trials published so far have reported the more common complications of prematurity as secondary outcomes and, subsequently, these have been taken into account by meta-analyses. Nonetheless, these were mere statistical investigations without any focus on the physiopathological or biological plausibility of their results. In our opinion, it is extremely important to couple data coming from randomized trials with the relevant biological and physiopathological knowledge in order to reduce misinterpretations and to avoid creating hopes that can hardly be confirmed. Given the clinical equivalence of bovine surfactants of different extraction method (minced or lung lavage) [8], and their similar biochemical/pharmacological features [7, 9], we decided to perform a pragmatic meta-analyses of porcine versus bovine surfactants with regard to extra-pulmonary outcomes, following the same pragmatic design adopted for the analysis of respiratory outcomes [7]. To provide more useful data we also comprehensively reviewed the available physiopathological informations regarding the possible links between surfactant replacement and neonatal non-respiratory outcomes.

## Methods

### Protocol

Prior to commencing the search, a systematic review protocol was agreed to determine the databases to be searched, search modality, eligibility criteria, data extraction/aggregation methodology, timing of meetings and methods for dispute resolution in case of disagreement. Following the agreement, this review was registered in the international prospective register of systematic review (PROSPERO n.CRD42018100906). Regular meetings between the authors were scheduled and the Preferred Reporting Items for Systematic Reviews and Meta-Analyses (PRISMA) guidelines were followed through the entire project [16]. This study has no funding.

### Eligibility criteria

The systematic review included randomized controlled trials fulfilling the following criteria: 1) published as full articles; 2) enrolled preterm neonates (gestational age < 37 weeks) with clinical and/or radiological evidence of RDS; 3) compared porcine and bovine surfactants (irrespective of their preparation method), and 4) reported at least one of the selected extra-pulmonary outcomes (see below). Studies were included in the meta-analysis, if they compared surfactants internationally available on the market. Since early selective surfactant treatment is currently advised by international guidelines [2, 3], we did not consider trials on surfactant prophylaxis. No language or year restrictions were applied. We excluded “grey” literature, unpublished or non-peer reviewed reports.

### Information sources, search strategy and study selection

These details are reported in the Additional file 1.

### Data collection process

We used a data extraction sheet based on the Cochrane Consumers and Communication Review Group’s data extraction template that had been already used in our previous work [7]. Data from included trials were extracted independently by the two authors and then cross-verified. Discrepancies were resolved through discussion between the two reviewers. Where further clarifications were needed or when data could not be statistically aggregated authors were contacted to provide clarification and/or raw data. At least two emails were sent to authors 2 weeks apart.

### Data items

Data collected included study design, number of enrolled patients, prenatal steroid, mean gestational age, inclusion and exclusion criteria, surfactant type and doses, outcomes, and variables used to assess study quality. The extra-pulmonary outcomes were: 1) length of stay in neonatal intensive care units (NICU); 2) hemodynamically significant patent *ductus arteriosus* (PDA), defined with any of the previously published criteria [17]; 3) intra-ventricular hemorrhage (IVH) of any grade according to Papile’s classification [18]; 4) Grade III-IV IVH according to Papile’s classification [18]; 5) necrotizing entero-colitis (NEC) of any stage, according to Bell’s classification [19]; 6) retinopathy of prematurity (ROP) of any stage according to current international classification [20]; The choice of focusing on newborn outcomes mainly irrespective of their stage/grade was pragmatic. In fact, these are relatively rare outcomes and trials seldom report them homogenously, reflecting real life differences in their bedside definition and management. Thus, our pragmatic choice allowed to have larger datasets to analyze.

### Assessment of risk of Bias

The Cochrane Risk of Bias assessment tool was used to evaluate quality of reviewed studies [21]. Two reviewers (DDL, SF) independently assessed the risk of bias for each trial, including: 1) selection bias (inadequate random sequence generation, failure to conceal treatment allocation); 2) performance bias (inadequate blinding of patients and investigators/personnel); 3) detection bias (failure to adequately blind the outcome assessors); 4) attrition bias (incomplete outcome data evaluation and failure to follow intention-to-treat analysis); 5) reporting bias (selective outcome reporting); 6) any other bias and any potential conflict of interest. Each item was assessed as at “low” or “high risk” of bias, or unclear (when the authors were unable to determine, on the basis of the available information). Discrepancies were resolved through discussion between the two reviewers. The presence of publication bias was explored, as recently suggested [22], through multiple methods including visual assessment of Funnel plot, Egger regression and the searching in trials’ registries and conference proceedings, as described above. More details are available in the Additional file 1.

### Summary measures and synthesis of results

Our previous meta-analysis about respiratory outcomes demonstrated similar results when we compared 100 mg/kg bovine surfactants versus 200 mg/kg or versus any dose of poractant-α (i.e.: pooling the data from all study arms in which poractant-α was administered, irrespective of the dosage used) [7]. Results of these comparisons were similar because only three trials randomized small newborn populations to receive a lower poractant-α dose: thus, these trials had a minor impact on the analyses. Moreover, non-respiratory outcomes are not always reported in each trial and this may reduce the sample size. Therefore, we pragmatically decided here to compare 100 mg/kg bovine surfactants versus any dose of poractant-α in order to have larger datasets. For the same reasons we did not perform a meta-analysis comparing 100 mg/kg poractant-α versus 100 mg/kg bovine surfactants, as this would have been unreliable, given the low number of patients. Outcomes were analyzed using weighted average odds ratios (OR) or mean difference and 95% confidence interval (95% CI), as appropriate. We used the DerSimonian-Laird random-effects or the continuous random-effects models, for binary and continuous outcomes, respectively. Such approach is more conservative than the fixed-effects model, as it assumes the presence of heterogeneity among aggregated studies, based on the assumption that the studies considered are estimating different underlying effect sizes [23]. Consistency across the studies was evaluated using the *I*^2^ statistic (variation in ORs attributable to heterogeneity) and performing a χ^2^ test for heterogeneity; an *I*^2^ value greater than 50% was considered as indicative of substantial heterogeneity. When no significant heterogeneity was found, we also repeated the analysis using fixed-effects or continuous fixed-effects inverse variance method, for binary and continuous outcomes, respectively.

### Additional analyses

Prenatal steroid prophylaxis and gestational age (GA) can be confounders, as they might influence the incidence of some of our outcomes [24]. Furthermore, since trials have been published across several years (from 1995 to 2017), steroids have been variously administered in the enrolled populations. Thus, when results of the meta-analysis were statistically significant, we performed two random-effects model meta-regressions [25] and we inserted as covariates: 1) prenatal steroids (as % of neonates treated in each study), and 2) the mean GA (in weeks) of each study population. We only used one covariate for each model to reduce false positive conclusions and we expressed results using coefficients (and 95%CI) [25]. All statistics were performed with Open-MetaAnalyst 10.1 [26] and Meta-essentials [27].

Finally, as further additional analysis, we aimed to understand why, from a physiopathological standpoint, surfactant replacement therapy (with any surfactant) would be able to improve extra-pulmonary outcomes, as this is actually debated [14, 15]. Thus, we performed a comprehensive review of possible physiopathological and biological mechanisms by which surfactant could influence extra-pulmonary outcomes. To do this, we searched information in the studies identified through the search strategy described above. Additionally, we also searched PubMed, using key words and/or MeSH terms as described in the Additional file 1, looking for animal or human translational investigations on these mechanisms.

## Results

**Fig. 1:**
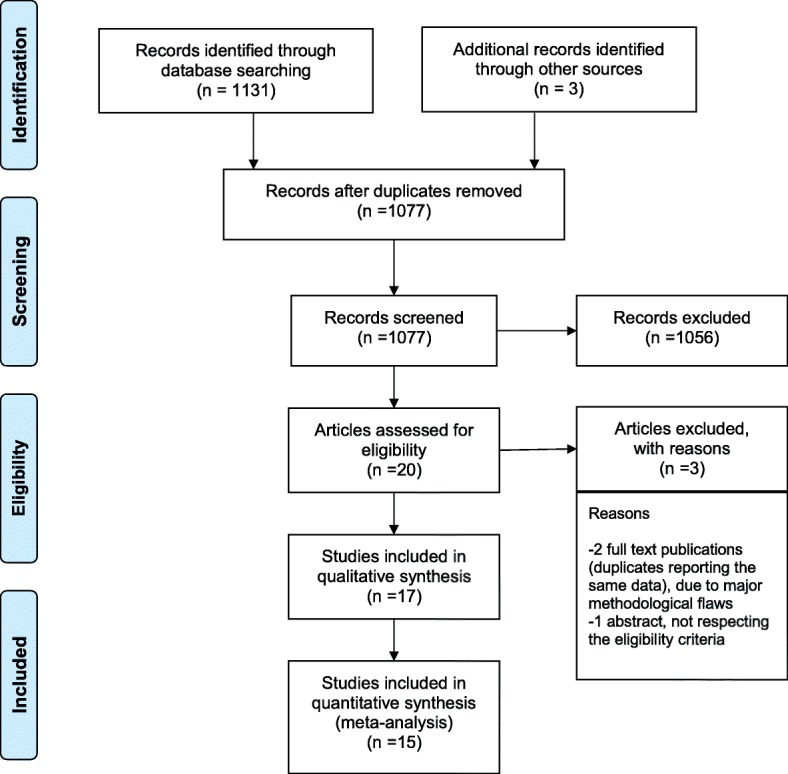
Flow chart of the review and meta-analysis. Randomized controlled trials published as full articles, enrolling preterm neonates (gestational age < 37 weeks) with clinical and/or radiological evidence of RDS, comparing porcine and bovine surfactants (irrespective of their preparation method) and reporting at least one extra-pulmonary outcomes were included in the systematic review. The excluded studies were two full text duplicates reporting the same data with major methodological flaws and one conference abstract which did not respect the eligibility criteria. Details of excluded studies are available in [7]

**Fig. 2:**
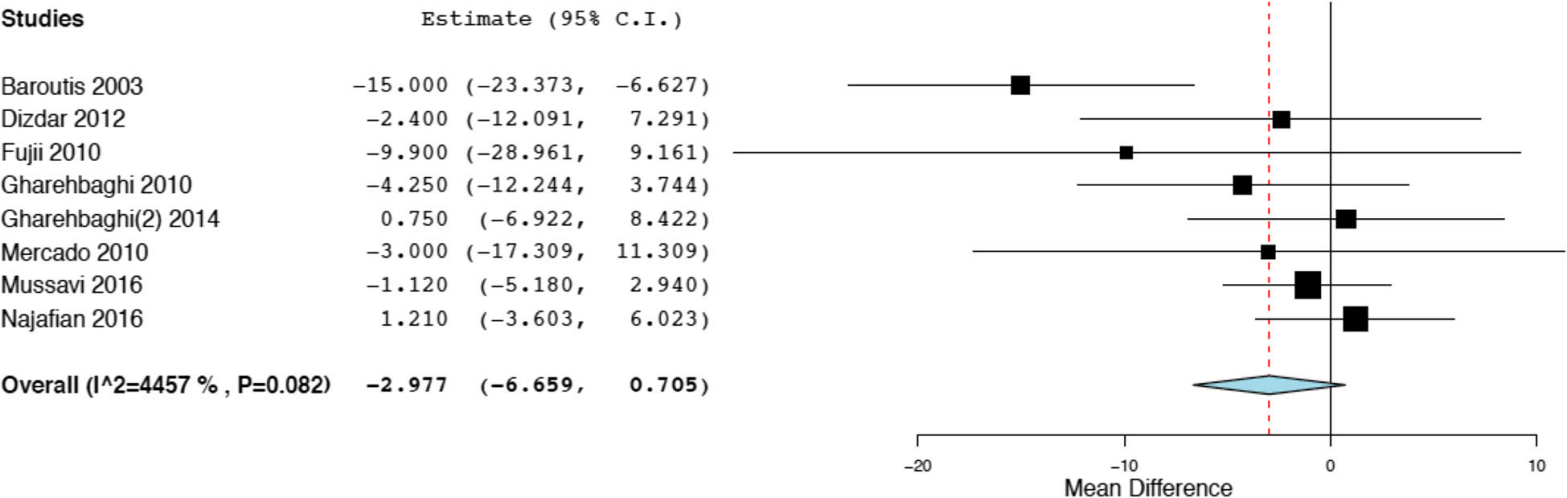
Comparisons poractant-α vs bovine surfactants for NICU length of stay. The panel (855 patients) illustrates meta-analyses of any dose of poractant-α vs bovine surfactants with random-effects model. Poractant-α and bovine surfactants are considered as treatment and control arm, respectively; mean differences (95%CI) in NICU stay are reported (in days). Squares and horizontal lines represent mean differences in NICU stay (expressed in days) and their 95%CI, respectively. The location of the diamond and its width represent the pooled estimated effect size and its 95%CI, respectively

**Fig. 3:**
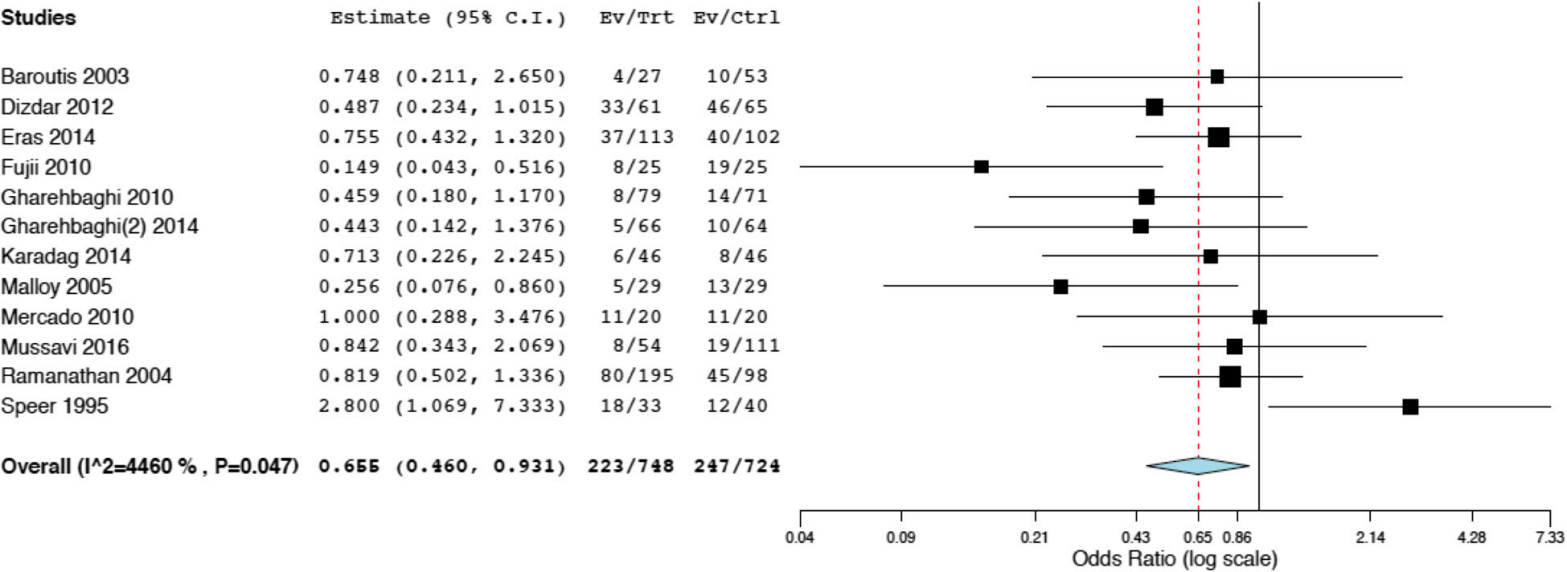
Comparisons poractant-α vs bovine surfactants for PDA. The panel (1472 patients) illustrates meta-analyses of any dose of poractant-α vs bovine surfactants with random-effects model. Poractant-α and bovine surfactants are considered as treatment (Trt) and control (Ctrl) arm, respectively; events (Ev) per arm and odds ratio (95%CI) are reported. Squares and horizontal lines represent odds ratios and their 95%CI, respectively. The location of the diamond and its width represent the pooled estimated effect size and its 95%CI, respectively

**Fig. 4:**
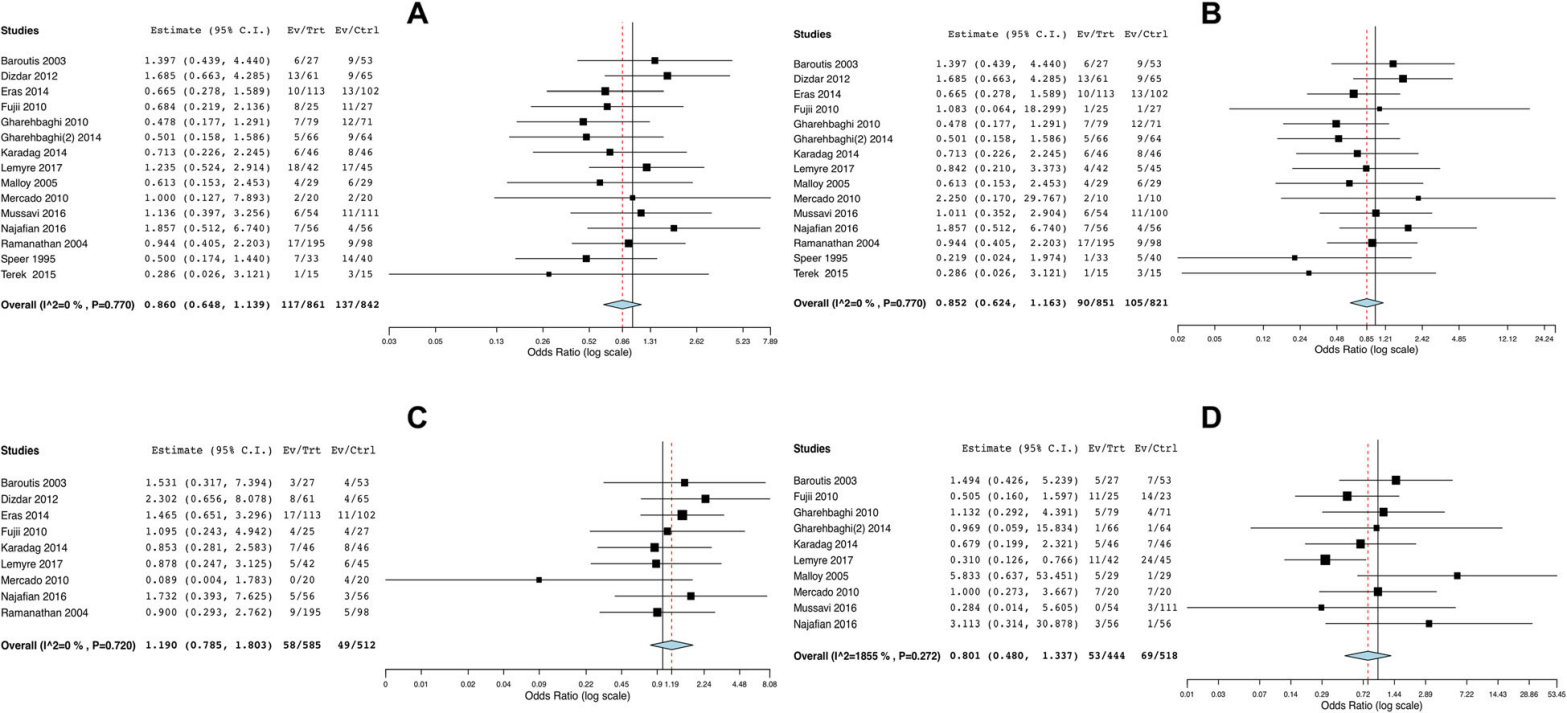
Comparisons poractant-α vs bovine surfactants for IVH (**a**-**b**), NEC(**c**) and ROP (**d**). **a** and **b** illustrate meta-analyses of any dose of poractant-α vs bovine surfactants for IVH of any grade (1703 neonates) and for grade III-IV IVH (1672 neonates), respectively. **c** and **d** illustrate NEC and ROP of any stage (1097 and 962 neonates, respectively). Poractant-α and bovine surfactants are considered as treatment (Trt) and control (Ctrl) arm, respectively; events (Ev) per arm and odds ratio (95%CI) are reported. Squares and horizontal lines represent odds ratios and their 95%CI, respectively. The location of the diamond and its width represent the pooled estimated effect size and its 95%CI, respectively. All analyses have been performed with random-effects model

Fig.1 illustrates the project flow-chart: we included 17 studies in the systematic review [28–45] and we excluded two papers from the meta-analysis, as they investigated two non-internationally marketed porcine surfactants [43, 44]. Compared to our previous meta-analysis on respiratory endpoints, there was one more study reporting on the extra-pulmonary outcomes [45]. This study did not report the exact dose of surfactant [45]. Moreover, Fujii et al. reported hemodynamically significant PDA in one paper and other extra-pulmonary outcomes in a second manuscript [30, 31]. Data were extracted from these two distinct papers, as appropriate: since the enrolled population was the same, they were considered as a single study and 15 studies were finally included in the meta-analysis. The other trials have been already described [7] and their characteristics are summarized in the Additional file 1. A total of 1721 neonates were enrolled in the 15 trials. Evaluation of biases is reported in the Additional file 1. The studies performed mostly well in completeness of outcome analysis and reporting, but generally suffered from performance bias due to imperfect blinding for interventions and outcome assessments (apart from one [40]). The methods of randomization and allocation concealment were unclear for the majority of studies. There seemed to be no significant Funnel plot asymmetry and, consistently, we found no unpublished project focusing on our extra-pulmonary outcomes in any trial registries. Fig.2 shows that there is no significant difference between poractant-α and bovine surfactants in terms of NICU stay (*p* = 0.113). Heterogeneity is at the border of significance. We also repeated the analysis using continuous fixed-effects model and found similar results (mean difference (days): -2.07; 95%CI: − 4.51-0.36; *p* = 0.094). Poractant-α is associated with significantly lower incidence of hemodynamically significant PDA (Fig.3: *p* = 0.018) and studies showed a statistically significant heterogeneity. We performed meta-regressions as additional analyses and the effect size of poractant-α on PDA did not result significantly associated neither with gestational age (coefficient: 0.079, 95%CI:-0.18–0.34, *p* = 0.554), nor with prenatal steroids (coefficient: -0.009, 95%CI: − 0.03-0.009, *p* = 0.323). There are no significant differences between poractant-α and bovine surfactants in terms of IVH of any grade (Fig.4a: *p* = 0.293) or grade III-IV IVH (Fig.4b: *p* = 0.313), or in terms of NEC (Fig.4c: *p* = 0.412) and ROP (Fig.4d: *p* = 0.396). There is a significant heterogeneity only for ROP. We repeated the meta-analyses for IVH and NEC using fixed effects-inverse variance method obtaining similar results (IVH any grade: OR: 0.86, 95%CI: 0.65–1.14, *p* = 0.293; grade III-IV IVH: OR: 0.85, 95%CI: 0.62–1.16, *p* = 0.313; NEC: OR: 1.19, 95%CI: 0.78–1.80, *p* = 0.412). Table 1 shows possible mechanisms by which surfactant could influence extra-pulmonary outcomes [34, 46–69]. Of note, a physiopathological link between surfactant administration and non-respiratory neonatal outcomes could have been demonstrated only for hemodynamically significant PDA.

**Table 1:** Mechanisms linking surfactant replacement to non-respiratory neonatal outcomes, as per animal or human translational investigations

	Physiopathological mechanisms	Hypothesized	Confirmed
PDA	↓ oxygen and ROS exposure		[46–49]
	↓ Prostaglandins synthesis		[50, 51]
	↓ PVR		[46, 47, 52–54]
IVH	↓ PaCO2 and CBF	[55–57]	
	↓ PDA	[53, 54]	
	Better peripheral perfusion	[34, 42]	
	Improved cerebral oxygenation	[58]	
ROP	↓ oxygen and ROS exposure	[49, 59–63]	
NEC	↓ oxygen and ROS exposure	[49, 64]	
	Better peripheral perfusion	[34, 42]	
	Earlier progression to full enteral feeding	[65–68]	

More details in the text. *Abbreviations*: *ROS* Reactive oxygen species, *PVR* Pulmonary vascular resistances, *CBF* Cerebral blood flow, *PaCO2* Arterial partial pressure of CO2, *PDA* Patent *ductus arteriosus*, *IVH* Intraventricular hemorrhage, *ROP* Retinopathy of prematurity, *NEC* Necrotizing enterocolitis

## Discussion

### Summary of evidence

Our meta-analysis compared for the first time the effects of poractant-α with all bovine surfactants on extra-pulmonary outcomes, using an aggregate sample larger than the ones used in previous meta-analyses [8, 70]. This has been possible by including recently published studies and using a particular data aggregation. This latter is fully justified by the previously demonstrated clinical equivalence of different bovine surfactants and their similar biochemical and pharmacological features [7–10]. To summarize, our results show a significantly reduced incidence of hemodynamically significant PDA in neonates treated with poractant-α, compared to those treated with bovine surfactants. The effect size of poractant-α on PDA is not influenced by antenatal steroids or gestational age. The clinical relevance of PDA in preterm neonates is currently debated, thus it is not clear if the surfactant effect on PDA incidence can actually represent a clinically important benefit. All other outcomes (NICU stay, NEC, ROP and IVH) are similar between patients treated with poractant-α and other surfactants. Heterogeneity is significant for PDA, ROP and nearly significant for NICU stay. This is likely due to: 1) the different criteria used in the studies for the diagnosis of hemodynamically significant PDA [17]; 2) the different screening policies for ROP, and PDA [17, 71]; 3) the influence of several different factors on NICU stay. This is an extremely complex variable that may be affected also by clinical problems occurring much later than surfactant administration, as well as logistic, social and psychological factors. However, all these issues contribute to actual NICU care and we aimed to perform a pragmatic meta-analysis focusing on the available data in the context of the real NICU care, rather than in a controlled experimental setting.

These results are only partially similar to those of the earlier Cochrane meta-analysis [8] but they are also stronger. In fact, compared to the previous work we: 1) included five more trials (accounting for ≈600 neonates); 2) used a trials aggregation based on the current best knowledge, that is, on the clinical equivalence of bovine surfactant [8]; 3) analyzed the effect of possible confounders, such as antenatal steroids or gestational age, and finally, 4) reviewed the possible physiopathological mechanisms linking surfactant replacement and extra-pulmonary outcomes. In fact, ours was intended to be not only a statistical work but also a multidisciplinary project coupling clinical outcomes with their physiopathological plausibility.

Interestingly, a convincing physiopathological link seems evident only between PDA and the choice of a particular surfactant. A surfactant more efficient from a biophysical point of view may be able to increase lung compliance and recruit alveoli more quickly, thus allowing to reduce supplemental oxygen. The oxygen exposure is important for the PDA closure [72] and the reduction in circulating reactive oxygen species may crosstalk with the inflammation pathway and contribute to reduce the synthesis of prostaglandins, which are also crucial for ductal patency [72]. Finally, a more efficient surfactant may quickly lower pulmonary vascular resistance through the alveolar recruitment and the increment in alveolar oxygen tension: this will facilitate the inversion of blood flow through the *ductus arteriosus* [73]. We cannot clarify if the effect of poractant-α on PDA incidence is due to his different phospholipid/protein profile or to the higher concentration allowing the use of higher doses, as already specified in our previous work on respiratory outcomes [7]. However, from a clinically pragmatic point of view this question is useless because: 1) pharmacokinetic and clinical data show that the high dose poractant-α has to be preferred over the low dose regimen, as it provides longer half-life, less re-treatments, better response in terms of oxygenation and improved respiratory outcomes [7, 74, 75]; 2) high dose regimens are unfeasible with bovine surfactants given their lower concentration and higher viscosity [9, 10]. These characteristics of bovine surfactants might negatively impact on hemodynamics and peripheral perfusion, yet a low doses regimen [34], while larger doses could cause tube occlusions, ventilation troubles and lung edema, potentially increasing the need for more aggressive ventilation.

Conversely, there are no convincing physiopathological data allowing to assume that one surfactant should be better than others in reducing the incidence of extra-pulmonary outcomes other than PDA. Some mechanisms have been hypothesized (Table 1) but they have not been confirmed for several reasons. For instance, surfactant administration and IVH incidence could theoretically be linked by the reduction of cerebral blood flow due to improved oxygenation and lower level of PaCO_2_. However, significant hypercarbia and hypoxia are rarely seen in the natural course of RDS (especially if antenatal steroids are given and early CPAP is efficiently provided) [76]: thus, when CPAP fails and surfactant is optimally administered, a significant change of PaCO_2_ is unlikely to be observed [76]. A reduced incidence of hemodynamically significant PDA could also theoretically influence the occurrence of IVH, but there are no convincing data linking PDA to IVH occurrence [77]. The achievement of a better peripheral perfusion in the peri-administration period has also been linked to IVH and NEC reduced incidence, however available data are not consistent and not all variables related to peripheral perfusion coherently change during surfactant dosing [34, 42]. A reduction in oxidative damage has been hypothesized as link between surfactant replacement and reduced incidence of ROP and NEC. However, all these disorders are often developing well after the early neonatal period, following genetic predisposition, long oxygen exposure and/or several cofactors (nutritional troubles, infections, transfusions, other pro-inflammatory triggers) [78, 79]. Therefore, it seems unlikely that a single drug administration, performed several days/weeks earlier, in another organ, could interfere with such a complex physiopathology. It is important to remind that the incidence of these extra-pulmonary neonatal outcomes have never been changed with the use of any surfactant, neither in the early trials conducted decades ago [80, 81], nor in the more recent ones, as acknowledged by the American Academy of Pediatrics both in 2008 and 2014 guidelines [3, 59]. Moreover, these outcomes were not changed when comparing surfactant therapy with prophylaxis and surfactant replacement increases survival rates without a change in the incidence of long-term neuro-developmental injury [45, 59]. Therefore, the lack of clear physiopathological plausibility and the preponderance of evidence suggest that the incidence of these outcomes is not significantly affected by any type of surfactant therapy. This is an important information in order to avoid false hopes and to help designing more physiopathologically solid trials. In fact, other questions remain open about surfactant replacement therapy and future investigation plans should concentrate on these, rather than asking surfactants to magically reduce various complications of prematurity.

### Limitations

We have chosen outcomes easily defined to allow data aggregation, albeit there are differences in outcomes’ diagnostic criteria amongst trials. However, we decided to use this pragmatic approach, as differences reflect the reality of NICU care. This approach helps to have larger patient populations and, when it obtains any positive results, they are very likely to be robust and generalizable [82]. Thus, the pragmatic design is known to be more appropriate when evaluating interventions which are refinements of current care [83]: as surfactant is already a cornerstone of neonatal critical care, this seemed the best strategy to apply. Studied populations were relatively small and the quality of the studies varied: potentially relevant biases were detected in almost every trial and this can impact on our findings, although they represent the best currently available evidence on the topic. We did not perform an individual patient meta-analysis, but rather meta-regressions and therefore some results might be subjected to the limitations of this technique or some confounders may have been missed. Finally, since the majority of studies investigated poractant-α at 200 mg/kg, we cannot draw any conclusion about the low dose poractant-α regimen. However, given the known clinical and pharmacokinetic advantages of 200 mg/kg dose of poractant-α [7, 74, 75], it seems unethical to design a trial only to verify if the higher dose or the biochemical composition is responsible for the clinical effects. Conversely, trials with high doses of bovine surfactant would be almost technically impossible [9, 10, 34]. In fact, as bovine surfactants are less concentrated and more viscous, higher doses can cause lung edema requiring more aggressive ventilation. Also, hemodynamic impairment has already been described with usual doses of bovine surfactants [33]. For these reasons, also the more recent animal studies only investigated bovine surfactants at their licensed dose [57].

## Conclusions

Poractant-α is associated with lower incidence of hemodynamically significant PDA than bovine surfactants; this effect is not influenced by gestational age or the use of prenatal steroids. There are no differences between porcine and bovine surfactants in terms of other extra-pulmonary outcomes. Since there is no physiopathological plausibility linking surfactant replacement to non-respiratory outcomes other than PDA, these endpoints should not be used in future trials.

## Supplementary information

**Additional file 1.** Porcine versus bovine surfactant therapy for RDS in preterm neonates: pragmatic meta-analysis and review of physiopathological plausibility of the effects on non-respiratory outcomes.

## Data Availability

All raw data generated or analyzed during this study are included in this published article [and its supplementary information files].

## Abbreviations

BPD: Broncho-pulmonary dysplasia
CPAP: Continuous positive airway pressure
GA: Gestational age
IVH: Intra-ventricular hemorrhage
NEC: Necrotizing enterocolitis
NICU: Neonatal intensive care unit
OR: Odds ratios
PDA: Hemodynamically significant patent *ductus arteriosus*
PRISMA: Preferred Reporting Items for Systematic Reviews and Meta-Analyses
RDS: Respiratory distress syndrome
ROP: Retinopathy of prematurity
