# Supplementary material for: Porcine versus bovine surfactant therapy for RDS in preterm neonates: pragmatic meta-analysis and review of physiopathological plausibility of the effects on extra-pulmonary outcomes
Source: Respir Res. 2020 Jan 7;21:8. doi: 10.1186/s12931-019-1267-8 (PMC6947871; doi:10.1186/s12931-019-1267-8)
Supplement: Supplementary file 1 — Additional file 1. Porcine versus bovine surfactant therapy for RDS in preterm neonates: pragmatic meta-analysis and review of physiopathological plausibility of the effects on non-respiratory outcomes. [file 12931_2019_1267_MOESM1_ESM.docx]

**ADDITIONAL ONLINE SUPPLEMENTARY MATERIAL**

**for**

**“Porcine *versus* bovine surfactant therapy for RDS in preterm neonates: pragmatic meta-analysis and review of physiopathological plausibility of the effects on non-respiratory outcomes”**

**Silvia Foligno** (MD) and **Daniele De Luca** (MD, PhD)

**eMethods 1.** Information sources, search strategy and study selection **Pag.2**

**eMethods 2.** Additional search criteria for the physiopathological plausibility. **Pag.2-5**

**eTable 1.** Characteristics of studies included in the systematic review and in the meta-analysis. **Pag.6-8**

**eFigure 1.** Results of bias assessment. **Pag.9**

**eReferences Pag.10-12**

**eMethods 1. *Information sources, search strategy and study selection***

We conducted a literature search (on Dec, 2018) of the following databases: AMED, Trip Database, BNI, CINAHL, EMBASE, HBE, HMIC, Medline, PsycINFO, PubMed, using the NICE National Institute for Healthcare Excellence Healthcare Databases Advanced Search portal. We used the following as key words and/or MeSH terms: “treatment”, “bovine lipid extract surfactant”, “BLES”, “beractant”, “survanta”, “surfacen”, “surfactant-TA”, “surfacten”, “bovactant”, “alveofact”, “calfactant”, “infasurf”, “poractant alfa”, “curosurf”, “newfactant”. We searched the abstract archives of the Pediatric Academy Society and European Society of Pediatric Research meetings; we searched the [clinicaltrials.gov](http://clinicaltrials.gov/) and IRSCTN registries. We also hand-searched references cited in the studies identified through the initial search, review articles on the subject and the authors’ personal archives. Finally, we contacted experts in the field and letters commenting the trials have also been reviewed. We used the following string: (treatment AND ((((bovine AND lipid) AND extract) AND surfactant) OR BLES OR beractant OR survanta OR surfacen OR surfactant-TA OR surfacten OR bovactant OR alveofact OR calfactant OR infasurf OR poractant alfa OR curosurf , OR newfactant)).ti,ab

Details of all studies retrieved were included in a database, removing duplicates. All authors reviewed abstracts, and (where necessary) full text of the remaining articles, excluding those not meeting the eligibility criteria

**eMethods 2. Additional search criteria for the physiopathological plausibility.** The following key words and/or MeSH terms were inputted in PubMed to search for informations about physiopathological mechanisms possibly linking surfactant with non-respiratory outcomes.

**For NICU stay:**

("surface-active agents"[Pharmacological Action] OR "pulmonary surfactants"[Pharmacological Action] OR "surface-active agents"[MeSH Terms] OR ("surface-active"[All Fields] AND "agents"[All Fields]) OR "surface-active agents"[All Fields] OR "surfactant"[All Fields] OR "pulmonary surfactants"[MeSH Terms] OR ("pulmonary"[All Fields] AND "surfactants"[All Fields]) OR "pulmonary surfactants"[All Fields]) AND ("intensive care units, neonatal"[MeSH Terms] OR ("intensive"[All Fields] AND "care"[All Fields] AND "units"[All Fields] AND "neonatal"[All Fields]) OR "neonatal intensive care units"[All Fields] OR "nicu"[All Fields]) AND stay[All Fields]

**For PDA:**

("surface-active agents"[Pharmacological Action] OR "pulmonary surfactants"[Pharmacological Action] OR "surface-active agents"[MeSH Terms] OR ("surface-active"[All Fields] AND "agents"[All Fields]) OR "surface-active agents"[All Fields] OR "surfactant"[All Fields] OR "pulmonary surfactants"[MeSH Terms] OR ("pulmonary"[All Fields] AND "surfactants"[All Fields]) OR "pulmonary surfactants"[All Fields]) AND PDA[All Fields]

and

("surface-active agents"[Pharmacological Action] OR "pulmonary surfactants"[Pharmacological Action] OR "surface-active agents"[MeSH Terms] OR ("surface-active"[All Fields] AND "agents"[All Fields]) OR "surface-active agents"[All Fields] OR "surfactant"[All Fields] OR "pulmonary surfactants"[MeSH Terms] OR ("pulmonary"[All Fields] AND "surfactants"[All Fields]) OR "pulmonary surfactants"[All Fields]) AND ("ductus arteriosus, patent"[MeSH Terms] OR ("ductus"[All Fields] AND "arteriosus"[All Fields] AND "patent"[All Fields]) OR "patent ductus arteriosus"[All Fields] OR ("patent"[All Fields] AND "ductus"[All Fields] AND "arteriosus"[All Fields]))

**For ROP:**

("surface-active agents"[Pharmacological Action] OR "pulmonary surfactants"[Pharmacological Action] OR "surface-active agents"[MeSH Terms] OR ("surface-active"[All Fields] AND "agents"[All Fields]) OR "surface-active agents"[All Fields] OR "surfactant"[All Fields] OR "pulmonary surfactants"[MeSH Terms] OR ("pulmonary"[All Fields] AND "surfactants"[All Fields]) OR "pulmonary surfactants"[All Fields]) AND ROP[All Fields]

and

("surface-active agents"[Pharmacological Action] OR "pulmonary surfactants"[Pharmacological Action] OR "surface-active agents"[MeSH Terms] OR ("surface-active"[All Fields] AND "agents"[All Fields]) OR "surface-active agents"[All Fields] OR "surfactant"[All Fields] OR "pulmonary surfactants"[MeSH Terms] OR ("pulmonary"[All Fields] AND "surfactants"[All Fields]) OR "pulmonary surfactants"[All Fields]) AND ("retinopathy of prematurity"[MeSH Terms] OR ("retinopathy"[All Fields] AND "prematurity"[All Fields]) OR "retinopathy of prematurity"[All Fields])

**For NEC:**

("surface-active agents"[Pharmacological Action] OR "pulmonary surfactants"[Pharmacological Action] OR "surface-active agents"[MeSH Terms] OR ("surface-active"[All Fields] AND "agents"[All Fields]) OR "surface-active agents"[All Fields] OR "surfactant"[All Fields] OR "pulmonary surfactants"[MeSH Terms] OR ("pulmonary"[All Fields] AND "surfactants"[All Fields]) OR "pulmonary surfactants"[All Fields]) AND NEC[All Fields]

and

("surface-active agents"[Pharmacological Action] OR "pulmonary surfactants"[Pharmacological Action] OR "surface-active agents"[MeSH Terms] OR ("surface-active"[All Fields] AND "agents"[All Fields]) OR "surface-active agents"[All Fields] OR "surfactant"[All Fields] OR "pulmonary surfactants"[MeSH Terms] OR ("pulmonary"[All Fields] AND "surfactants"[All Fields]) OR "pulmonary surfactants"[All Fields]) AND ("necrotising enterocolitis"[All Fields] OR "enterocolitis, necrotizing"[MeSH Terms] OR ("enterocolitis"[All Fields] AND "necrotizing"[All Fields]) OR "necrotizing enterocolitis"[All Fields] OR ("necrotizing"[All Fields] AND "enterocolitis"[All Fields]))

**For IVH:**

("surface-active agents"[Pharmacological Action] OR "pulmonary surfactants"[Pharmacological Action] OR "surface-active agents"[MeSH Terms] OR ("surface-active"[All Fields] AND "agents"[All Fields]) OR "surface-active agents"[All Fields] OR "surfactant"[All Fields] OR "pulmonary surfactants"[MeSH Terms] OR ("pulmonary"[All Fields] AND "surfactants"[All Fields]) OR "pulmonary surfactants"[All Fields]) AND intraventricular[All Fields] AND ("haemorrhage"[All Fields] OR "hemorrhage"[MeSH Terms] OR "hemorrhage"[All Fields])

and

("surface-active agents"[Pharmacological Action] OR "pulmonary surfactants"[Pharmacological Action] OR "surface-active agents"[MeSH Terms] OR ("surface-active"[All Fields] AND "agents"[All Fields]) OR "surface-active agents"[All Fields] OR "surfactant"[All Fields] OR "pulmonary surfactants"[MeSH Terms] OR ("pulmonary"[All Fields] AND "surfactants"[All Fields]) OR "pulmonary surfactants"[All Fields]) AND IVH[All Fields]

**eTable 1. Characteristics of studies included in the systematic review and in the meta-analysis.** Two studies were excluded from the pragmatic meta-analysis because they investigated the use of two non-internationally marketed porcine surfactants.

**§** All studies eligible for the review and meta-analysis enrolled preterm neonates with clinical and/or radiologic evidence of RDS: the additional inclusion criteria selected patients based on their clinical severity, gestational age and/or birth weight.

**#** refers to the mean gestational age of the whole trial population (as weighted mean gestational age of the different arms).

**^** RDS score is the so called “Downes' score”, which considers gestational age, respiratory rate, breath sounds on auscultation, grunting, retractions and FiO_2_ with possible scores ranging from 0 to 12. (9)

**†** Ramanathan’s study (14) trialled beractant versus high dose (200 mg/kg) or low dose (100 mg/kg) poractant-α. Thus, data from these arms were pooled together for the analysis, irrespective of the dosage used.

**n.a.:** indicates data not available (not described in the paper and not provided by the authors asked for)

Studies with an asterisk (*****) have a multicentre design.

**Abbreviations**: a/A: arterial/alveolar ratio; BPD: Broncho-pulmonary dysplasia; BW: birth weight; FiO_2_: inspired oxygen fraction; GA: gestational age; OI: oxygenation index (Paw x FiO_2_/PaO_2_); PaO_2_: arterial partial pressure of oxygen; Paw: mean airway pressure; RDS: respiratory distress syndrome; SatO_2_: peripheral arterial oxygen saturation.

| **Studies included in the meta-analysis** | | | | | | | | | |
| --- | --- | --- | --- | --- | --- | --- | --- | --- | --- |
| **Author/year (ref.)** | **Country** | **Comparison**  **(n° of patients)** | **Dose**  **(mg/kg)** | | **Additional inclusion criteria §** | | **Antenatal steroids (%/arm)** | **GA#**  **(weeks)** | **Outcomes** |
| Baroutis/2003 (1)  commented in (2) | Greece | Bovactant (27) *vs* Poractant-α (27) *vs* Beractant (26) | 100 *vs* 100 *vs* 100 | | BW≤2000g - FiO_2_>0.3  (within 24h of life) | | 33% - 26% - 31% | 29 ± 0.9 | PDA ROP  NEC  IVH (≥ II grade)  Length of hospital stay |
| Dizdar/2012 (3) | Turkey | Poractant-α (61)  *vs* Beractant (65) | 200 *vs* 100 | | FiO_2_≥0.30 (within 6h of life) | | 61% - 51% | 28 ± n.a. | IVH  PDA  NEC  Length of hospital stay |
| Fuji/2010* (4)  commented in (5) | USA | Poractant-α (25)  *vs* Beractant (27) | 200 *vs* 100 | | GA<30wks - Need for mechanical ventilation with Paw≥6 cmH_2_O and FiO_2_>30% (within 8h of life) | | 100% - 96% | 26.9 ± 1.6 | PDA  IVH  PVL  ROP  NEC  Length of hospital study |
| Gharehbaghi/2010* (6) | Iran | Poractant-α (79)  *vs* Beractant (71) | 200 *vs* 100 | | None | | 45% - 42% | 29.5 ± 2.8 | PDA  IVH (III-IV)  ROP  Length of hospital study |
| Gharehbaghi/2014* (7) | Iran | Poractant-α (66)  *vs* Bovactant (64) | 200 *vs* 100 | | GA≤32wks - FiO_2_>30%  (within 6h of life) | | 62% - 53% | 28.3 ± 2.1 | PDA  IVH  ROP  Length of hospital study |
| Karadag/2014 (8) | Turkey | Poractant-α (46)  *vs* Beractant (46) | 200 *vs* 100 | | GA≤32wks – Need for mechanical ventilation with Paw>7 cmH_2_O and FiO_2_>0.4 to have PaO_2_=70-80 mmHg (within 2h of life) | | 76% - 83% | 29.3 ± 1.8 | IVH  NEC  PDA  ROP  PLV  Length of hospital stay |
| Lemyre/2017* (9) | Canada | Poractant-α (42)  *vs* BLES (45) | 200 *vs* 135 | | GA<32wks (within 48h of life) - RDS score^ >8 or >1 of the following: 1)  FiO_2_≥0.4; 2) pH<7.20 and PaCO_2_> 60mmHg (persistent & associated with clinical distress) or 3) recurrent apnea | | 67% - 73% | 26.7 ± 1.9 | IVH (all grades)  PLV  NEC ( II grade or higher)  ROP ( all stages)  ROP stage III -IV |
| Malloy/2005 (10) | USA | Poractant-α (29)  *vs* Beractant (29) | 200 *vs* 100 | | Need for surfactant as per clinical judgment and unit policy | | 69% - 79% | 29.5 ± 3.2 | IVH III-IV grade  PLV PDA |
| Mercado/2010 (11) | USA | Poractant-α (20)  *vs* Beractant (20) | 200 *vs* 100 | | GA<30wks | | 95% - 95% | 26 ± 1.5 | PDA  ROP  IVH  NEC  Length of hospital stay |
| Mussavi/2016 (12) | Iran | Bovactant (49) *vs* Poractant-α (62) *vs* Beractant (54) | 100 *vs* 200 *vs* 100 | | Within 6h of life | | 92% - 91% - 87% | 31.6 ± 3.7 | PDA  IVH  ROP  Length of hospital stay |
| Najafian/2016* (13) | Iran | Poractant-α (56)  *vs* Beractant (56) | 100 *vs* 100 | | BW>750g - SatO_2_ between 85% and 96% - GA<35wks (within 6h of life) | | n.a. | 32.6 ± 3.3 | IVH  NEC  ROP  Length of hospital stay |
| Ramanathan/2004* † (14)  commented in (15) | USA | Hi-dose Poractant-α (99)  *vs* Low-dose Poractant-α (96) *vs* Beractant (98) | 200 *vs* 100 *vs* 100 | | BW between 750 and 1750g – Need for mechanical ventilation with FiO_2_≥0.30 or a/A≤33 (within 6h of life) | | 76% - 82% - 85% | 28.5 ± 2 | PDA NEC IVH grade III-IV  * ( outcome also infants ≤ 32) |
| Speer/1995* (16)  commented in (17) | Germany | Poractant-α (33)  *vs* Beractant (40) | 200 *vs* 100 | | BW between 700 and 1500g - Need for mechanical ventilation with FiO_2_≥0.40 (within 24h of life) | | 42% - 37% | 28.8 ± 2.2 | Intracerebral haemorrhage total  IVH grade III-IV  PDA |
| Terek/2015 (18)  commented in (19) | Turkey | Poractant-α (15)  *vs* Beractant (18) | 200 *vs* 100 | | GA between 26 and 36wks - FiO_2_/SpO_2_<0.22 or FiO_2_>0.4 or OI>7 (within 2h of life) | | 72% - 80% | 30.1 ± 3.3 | IVH  PLV |
| Eras 2014 (20) | Turkey | Poractant-α (15)  *vs* Beractant (18) | n.a | A fraction of inspired  oxygen (FiO_2_) of > 0.4 in the first 2 hour of life to maintain SpO2 levels between 85 and 92% | | n.a | | 28.5 ± 2.3 | PDA  IVH  NEC |
| **Studies not included in the meta-analysis** | | |  |  | |  | |  |  |
| **Author/year** | **Country** | **Comparison (patients)** | **Dose**  **(mg/kg)** | | **Additional inclusion criteria §** | | **Antenatal steroids (%/arm)** | **GA (weeks)** | **Reason for exclusion from meta-analysis** |
| Rebello/2014 (21) | Brazil | Butantan (154) *vs* Poractant-α or Beractant (173) | 100 *vs* 100 | | BW≤500g; postnatal age -  PaO_2_/FiO_2_≤175 (within 24h of life) | | 51% - 54% | 28 ± 2 | Butantan not commercially available worldwide.  Control arm treated with a mix of Poractant-α or Beractant: impossible to separate data |
| Sanchez-Mendiola/2005 (22) | Mexico | Surfacen (21) vs Beractant (23) | 100 *vs* 100 | | FiO_2_>0.5 and Paw>8 cmH_2_O to have PaO_2_>60 mmHg (within 8h of life) | | n.a. | 31 ± 3.3 | Surfacen not commercially available worldwide. |

**eFigure 1. Results of bias assessment: risk of bias for each trial (A) and publication bias (B).** Panel A shows the risk of bias evaluation performed with the Cochrane Risk of Bias assessment tool (23). Each item was assessed as at “low”(+, green squares) or “high risk” (-, red squares) of bias, or unclear (?, yellow squares), when the investigators were unable to determine, on the basis of the available data. Panel B shows Funnel plot. This is a scatter plot of the studies in a meta-analysis (blue dots) in a space defined by effect size on the incidence of haemodynamically significant patent *ductus arteriosus* (on the x-axis; scale displayed on top of the plot) and standard error (on the y-axis). It also presents the combined effect size (CES; green dot) with its confidence interval (black). The plot also shows a vertical line (also in red) that runs through the (adjusted) combined effect size and the corresponding lower and upper limits of the confidence interval (red diagonal lines). The diagonal hatched line represents the line generated by the Egger regression (*p*=0.09). (24) The adjusted combined effect size and accompanying confidence and prediction intervals in this plot represents the results of a trim-and-fill procedure as proposed by Duval and Tweedie. (25,26)

**
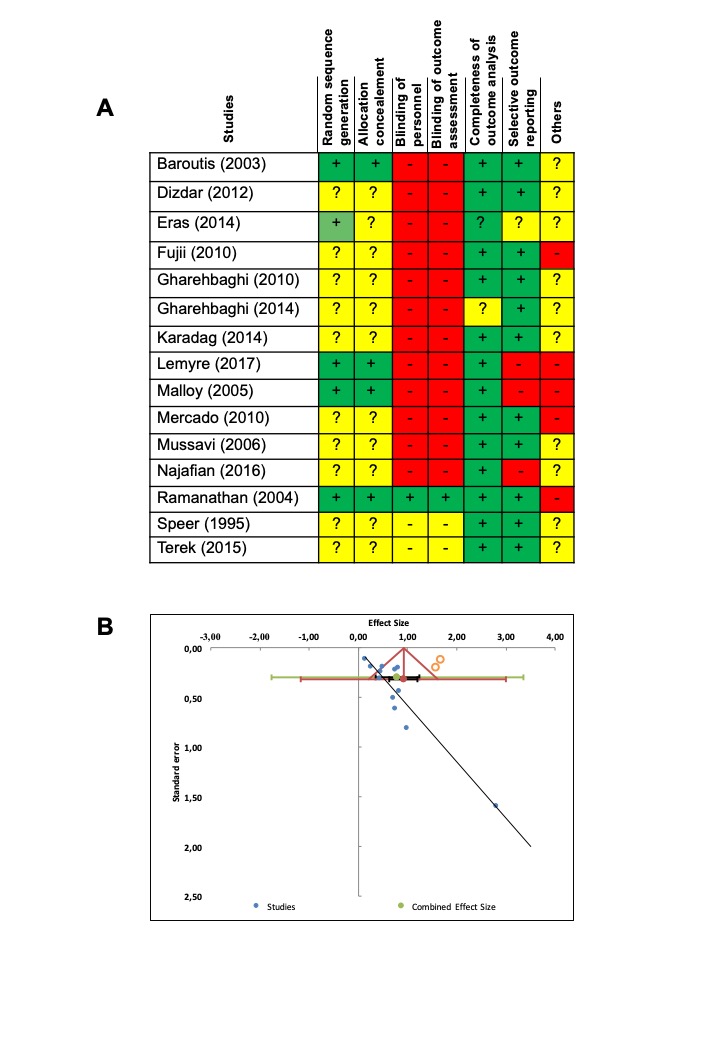
**

**eReferences**

1.[Baroutis G](https://www.ncbi.nlm.nih.gov/pubmed/?term=Baroutis%20G%5BAuthor%5D&cauthor=true&cauthor_uid=12709796), [Kaleyias J](https://www.ncbi.nlm.nih.gov/pubmed/?term=Kaleyias%20J%5BAuthor%5D&cauthor=true&cauthor_uid=12709796), [Liarou T](https://www.ncbi.nlm.nih.gov/pubmed/?term=Liarou%20T%5BAuthor%5D&cauthor=true&cauthor_uid=12709796), [Papathoma E](https://www.ncbi.nlm.nih.gov/pubmed/?term=Papathoma%20E%5BAuthor%5D&cauthor=true&cauthor_uid=12709796), [Hatzistamatiou Z](https://www.ncbi.nlm.nih.gov/pubmed/?term=Hatzistamatiou%20Z%5BAuthor%5D&cauthor=true&cauthor_uid=12709796), [Costalos C](https://www.ncbi.nlm.nih.gov/pubmed/?term=Costalos%20C%5BAuthor%5D&cauthor=true&cauthor_uid=12709796).Comparison of three treatment regimens of natural surfactant preparations in neonatal respiratory distress syndrome.

*Eur J Pediatr* 2003;**162**:476-80.

2. [Shalwitz RA](https://www.ncbi.nlm.nih.gov/pubmed/?term=Shalwitz%20RA%5BAuthor%5D&cauthor=true&cauthor_uid=14628142). Comparison of treatment regimens of natural surfactant preparations in neonatal respiratory distress syndrome. *Eur J Pediatr* 2004;**163**:126;

3. [Dizdar EA](https://www.ncbi.nlm.nih.gov/pubmed/?term=Dizdar%20EA%5BAuthor%5D&cauthor=true&cauthor_uid=22105435), [Sari FN](https://www.ncbi.nlm.nih.gov/pubmed/?term=Sari%20FN%5BAuthor%5D&cauthor=true&cauthor_uid=22105435), [Aydemir C](https://www.ncbi.nlm.nih.gov/pubmed/?term=Aydemir%20C%5BAuthor%5D&cauthor=true&cauthor_uid=22105435), et al. A randomized, controlled trial of poractant alfa versus beractant in the treatment of preterm infants with respiratory distress syndrome. *Am J Perinatol* 2012;**29**:95-100.

4. [Fujii AM](https://www.ncbi.nlm.nih.gov/pubmed/?term=Fujii%20AM%5BAuthor%5D&cauthor=true&cauthor_uid=20336076), [Patel SM](https://www.ncbi.nlm.nih.gov/pubmed/?term=Patel%20SM%5BAuthor%5D&cauthor=true&cauthor_uid=20336076), [Allen R](https://www.ncbi.nlm.nih.gov/pubmed/?term=Allen%20R%5BAuthor%5D&cauthor=true&cauthor_uid=20336076), [Doros G](https://www.ncbi.nlm.nih.gov/pubmed/?term=Doros%20G%5BAuthor%5D&cauthor=true&cauthor_uid=20336076), [Guo CY](https://www.ncbi.nlm.nih.gov/pubmed/?term=Guo%20CY%5BAuthor%5D&cauthor=true&cauthor_uid=20336076), [Testa S](https://www.ncbi.nlm.nih.gov/pubmed/?term=Testa%20S%5BAuthor%5D&cauthor=true&cauthor_uid=20336076). Poractant alfa and beractant treatment of very premature infants with respiratory distress syndrome. *J Perinatol* 2010;**30**:665-70.

5. Lutchman D. Have we been down this road before? *J Perinatol* 2010;**30**:698-9;

6. [Gharehbaghi MM](https://www.ncbi.nlm.nih.gov/pubmed/?term=Gharehbaghi%20MM%5BAuthor%5D&cauthor=true&cauthor_uid=20589459), [Sakha SH](https://www.ncbi.nlm.nih.gov/pubmed/?term=Sakha%20SH%5BAuthor%5D&cauthor=true&cauthor_uid=20589459), [Ghojazadeh M](https://www.ncbi.nlm.nih.gov/pubmed/?term=Ghojazadeh%20M%5BAuthor%5D&cauthor=true&cauthor_uid=20589459), [Firoozi F](https://www.ncbi.nlm.nih.gov/pubmed/?term=Firoozi%20F%5BAuthor%5D&cauthor=true&cauthor_uid=20589459). Complications among premature neonates treated with beractant and poractant alfa. *Indian J Pediatr* 2010;**77**:751-4.

7. [Gharehbaghi MM](https://www.ncbi.nlm.nih.gov/pubmed/?term=Gharehbaghi%20MM%5BAuthor%5D&cauthor=true&cauthor_uid=20589459), Yasrebi S. Comparing the Efficacy of two Natural Surfactants, Curosurf and Alveofact, in Treatment of Respiratory Distress Syndrome in Preterm Infants.

*Int J Women Health Reprod Sci* 2014;**2**:245-248.

8. [Karadag N](https://www.ncbi.nlm.nih.gov/pubmed/?term=Karadag%20N%5BAuthor%5D&cauthor=true&cauthor_uid=24566756), [Dilli D](https://www.ncbi.nlm.nih.gov/pubmed/?term=Dilli%20D%5BAuthor%5D&cauthor=true&cauthor_uid=24566756), [Zenciroglu A](https://www.ncbi.nlm.nih.gov/pubmed/?term=Zenciroglu%20A%5BAuthor%5D&cauthor=true&cauthor_uid=24566756), [Aydin B](https://www.ncbi.nlm.nih.gov/pubmed/?term=Aydin%20B%5BAuthor%5D&cauthor=true&cauthor_uid=24566756), [Beken S](https://www.ncbi.nlm.nih.gov/pubmed/?term=Beken%20S%5BAuthor%5D&cauthor=true&cauthor_uid=24566756), [Okumus N](https://www.ncbi.nlm.nih.gov/pubmed/?term=Okumus%20N%5BAuthor%5D&cauthor=true&cauthor_uid=24566756). Perfusion index variability in preterm infants treated with two different natural surfactants for respiratory distress syndrome. *Am J Perinatol* 2014;**31**:1015-22.

9. [Lemyre B](https://www.ncbi.nlm.nih.gov/pubmed/?term=Lemyre%20B%5BAuthor%5D&cauthor=true&cauthor_uid=28472058), [Fusch C](https://www.ncbi.nlm.nih.gov/pubmed/?term=Fusch%20C%5BAuthor%5D&cauthor=true&cauthor_uid=28472058), [Schmölzer GM](https://www.ncbi.nlm.nih.gov/pubmed/?term=Schm%C3%B6lzer%20GM%5BAuthor%5D&cauthor=true&cauthor_uid=28472058), et al. Poractant alfa versus bovine lipid extract surfactant for infants 24+0 to 31+6 weeks gestational age: A randomized controlled trial. *PLoS One* 2017;**12**:e0175922.

10. [Malloy CA](https://www.ncbi.nlm.nih.gov/pubmed/?term=Malloy%20CA%5BAuthor%5D&cauthor=true&cauthor_uid=16188788), [Nicoski P](https://www.ncbi.nlm.nih.gov/pubmed/?term=Nicoski%20P%5BAuthor%5D&cauthor=true&cauthor_uid=16188788), [Muraskas JK](https://www.ncbi.nlm.nih.gov/pubmed/?term=Muraskas%20JK%5BAuthor%5D&cauthor=true&cauthor_uid=16188788). A randomized trial comparing beractant and poractant treatment in neonatal respiratory distress syndrome. *Acta Paediatr* 2005;**94**:779-84.

11. [Mercado VV](https://www.ncbi.nlm.nih.gov/pubmed/?term=Mercado%20VV%5BAuthor%5D&cauthor=true&cauthor_uid=20586001), [Cristea I](https://www.ncbi.nlm.nih.gov/pubmed/?term=Cristea%20I%5BAuthor%5D&cauthor=true&cauthor_uid=20586001), [Ali N](https://www.ncbi.nlm.nih.gov/pubmed/?term=Ali%20N%5BAuthor%5D&cauthor=true&cauthor_uid=20586001), et al. Does surfactant type cause a differential proinflammatory response in preterm infants with respiratory distress syndrome? *Adv Ther* 2010;**27**:476-82.

12. [Mussavi M](https://www.ncbi.nlm.nih.gov/pubmed/?term=Mussavi%20M%5BAuthor%5D&cauthor=true&cauthor_uid=28203337), [Mirnia K](https://www.ncbi.nlm.nih.gov/pubmed/?term=Mirnia%20K%5BAuthor%5D&cauthor=true&cauthor_uid=28203337), [Asadollahi K](https://www.ncbi.nlm.nih.gov/pubmed/?term=Asadollahi%20K%5BAuthor%5D&cauthor=true&cauthor_uid=28203337). Comparison of the Efficacy of Three Natural Surfactants (Curosurf, Survanta, and Alveofact) in the Treatment of Respiratory Distress Syndrome Among Neonates: A Randomized Controlled Trial. *Iran J Pediatr* 2016;**26**:e5743.

13. Najafian B, Karimi-Sari H, Hossein Khosravi M, Nikjoo N, Amin S, Shohrati M. Comparison of efficacy and safety of two available natural surfactants in Iran, Curosurf and Survanta in treatment of neonatal respiratory distress syndrome: A randomized clinical trial. *Contemp Clin Trials Commun* 2016;**3**:55-59.

14. [Ramanathan R](https://www.ncbi.nlm.nih.gov/pubmed/?term=Ramanathan%20R%5BAuthor%5D&cauthor=true&cauthor_uid=15085492), [Rasmussen MR](https://www.ncbi.nlm.nih.gov/pubmed/?term=Rasmussen%20MR%5BAuthor%5D&cauthor=true&cauthor_uid=15085492), [Gerstmann DR](https://www.ncbi.nlm.nih.gov/pubmed/?term=Gerstmann%20DR%5BAuthor%5D&cauthor=true&cauthor_uid=15085492), [Finer N](https://www.ncbi.nlm.nih.gov/pubmed/?term=Finer%20N%5BAuthor%5D&cauthor=true&cauthor_uid=15085492), [Sekar K](https://www.ncbi.nlm.nih.gov/pubmed/?term=Sekar%20K%5BAuthor%5D&cauthor=true&cauthor_uid=15085492); [North American Study Group](https://www.ncbi.nlm.nih.gov/pubmed/?term=North%20American%20Study%20Group%5BCorporate%20Author%5D). A randomized, multicenter masked comparison trial of poractant alfa (Curosurf) versus beractant (Survanta) in the treatment of respiratory distress syndrome in preterm infants. *Am J Perinatol* 2004;**21**:109-19.

15. Bancalari E. On "A randomized, multicenter masked comparison trial of poractant alfa (Curosurf) versus beractant (Survanta) in the treatment of respiratory distress syndrome in preterm infants" (Am J Perinatol 2004;21:109-120). *Am J Perinatol* 2004;**21**:307-9.

16. Speer CP, Gefeller O, Groneck P, et al. [Randomised clinical trial of two treatment regimens of natural surfactant preparations in neonatal respiratory distress syndrome.](https://www.ncbi.nlm.nih.gov/pubmed/7743295) *Arch Dis Child Fetal Neonatal* Ed 1995;**72**:F8-13.

17. Logan S. Need to avoid bias in controlled trials. *Arch Dis Child Fetal Neonatal Ed* 1995;**73**:F121.

18. Terek D, Gonulal D, Koroglu OA, Yalaz M, Akisu M, Kultursay N. [Effects of Two Different Exogenous Surfactant Preparations on Serial Peripheral Perfusion Index and Tissue Carbon Monoxide Measurements in Preterm Infants with Severe Respiratory Distress Syndrome.](https://www.ncbi.nlm.nih.gov/pubmed/25603725) *Pediatr Neonatol* 2015;**56**:248-55.

19. Jeng MJ. Effects of Two Different Exogenous Surfactant Preparations on Serial Peripheral Perfusion Index and Tissue Carbon Monoxide Measurements in Preterm Infants with Severe Respiratory Distress Syndrome.

*Pediatr Neonatol* 2015;**56**:211-212.

20. Eras Z, Dizdar E, Kanmaz G, et al. Neurodevelopmental Outcomes of Very Low Birth Weight Preterm Infants Treated With Poractant Alfa versus Beractant for Respiratory Distress Syndrome. *Am J Perinatol* 2013;**31**:463-468. doi:10.1055/s-0033-1351659

21. Rebello CM, Precioso AR, Mascaretti RS, Grupo Colaborativo do Estudo Brasileiro Multicêntrico de Surfactante. A multicenter, randomized, double-blind trial of a new porcine surfactant in premature infants with respiratory distress syndrome. *Einstein (Sao Paulo)* 2014;**12**:397–404.

22. Sánchez-Mendiola M, Martínez-Nater OC, Herrera-Maldonado N, Ortega-Arroyo J. Estudio controlado del tratamiento de la enfermedad de membrana hialina del recién nacido pretérmino con surfactante pulmonar exógeno (porcino vs. bovino). *Gac Méd Méx* 2005;**4**:267-271. [article in Spanish]

23. Higgins JP, Altman DG, Gøtzsche PC, et al; Cochrane Bias Methods Group; Cochrane Statistical Methods Group. [The Cochrane Collaboration's tool for assessing risk of bias in randomised trials.](https://www.ncbi.nlm.nih.gov/pubmed/22008217) *BMJ* 2011;**343**:d5928.

24. [Sterne JA](https://www.ncbi.nlm.nih.gov/pubmed/?term=Sterne%20JA%5BAuthor%5D&cauthor=true&cauthor_uid=21784880), [Sutton AJ](https://www.ncbi.nlm.nih.gov/pubmed/?term=Sutton%20AJ%5BAuthor%5D&cauthor=true&cauthor_uid=21784880), [Ioannidis JP](https://www.ncbi.nlm.nih.gov/pubmed/?term=Ioannidis%20JP%5BAuthor%5D&cauthor=true&cauthor_uid=21784880), et al. Recommendations for examining and interpreting funnel plot asymmetry in meta-analyses of randomised controlled trials. *BMJ* 2011;**343**:d4002.

25. Duval S, Tweedie R. A nonparametric "trim and fill" method of accounting for publication bias in meta-analysis. *J Am Stat Assoc* 2000;**95**: 89-98.

26. Duval S. Tweedie R. Trim and fill: A simple funnel-plot-based method of testing and adjusting for publication bias in meta-analysis. *Biometrics* 2000;**56**:455-463.
